# Tackling constipation in patients on high dose antipsychotics

**DOI:** 10.1192/bjo.2021.384

**Published:** 2021-06-18

**Authors:** Sandhya Eappen

**Affiliations:** Parklands Hospital

## Abstract

**Aims:**

The aim of this audit was to look into patients on high dose antipsychotics who had developed complications of constipation in the PICU setting .

**Background:**

Antipsychotics are usually used in the treatment of Schizophrenia and other psychotic illnesses. Drug such as Clozapine mainly has a higher risk profile due to gastrointestinal hypo motility. It could present as constipation, fecal impaction or a bowel obstruction and could even lead to death.

**Method:**

During ward rounds enquired on bowel habits and diet.

Physical examination of patients complaining of abdominal pain.

Screened notes in past to see how many patients complained of constipation and interventions suggested and used.I65.

**Result:**

3 of the 10 patients on PICU were on high dose antipsychotics and 2 of them had reported constipation. Of which one required daily review and vigorous treatment with laxatives and dietary changes.

Recommendation

Bristol stool chart introduced as part of care plan for all patients.

Teaching presentation of constipation and its treatment management was given to the PICU team.

Involving medical team early on for assessment and prophylactic laxatives prescription.

Liaison with the pantry team to include more options of fruits and vegetables into daily meal plan for patients.

Data and material handed over to next trainee to Re-audit and complete audit cycle.

**Conclusion:**

Appropriate prevention and early management of side effects can enhance the benefits of antipsychotics. Bowel function monitoring and the use of prophylactic laxatives for patient on high dose antipsychotics such as clozapine is advisable to prevent complications related to it.

